# Actin Crosslinking Toxins of Gram-Negative Bacteria

**DOI:** 10.3390/toxins1020123

**Published:** 2009-12-01

**Authors:** Karla J. F. Satchell

**Affiliations:** Feinberg School of Medicine, Northwestern University, 320 E. Superior Avenue, Chicago, IL 60611, USA; Email: k-satchell@northwestern.edu; Tel.: +1-312-503-2162; Fax: +1-312-503-1339

**Keywords:** bacterial protein toxin, *Vibrio*, MARTX, VgrG, actin, ACD

## Abstract

Actin crosslinking toxins produced by Gram-negative bacteria represent a small but unique class of bacterial protein toxins. For each of these toxins, a discrete actin crosslinking domain (ACD) that is a distant member of the ATP-dependent glutamine synthetase family of protein ligases is translocated to the eukaryotic cell cytosol. This domain then incorporates a glutamate-lysine crosslink between actin monomers, resulting in destruction of the actin cytoskeleton. Recent studies argue that the function of these toxins during infection is not destruction of epithelial layers, but rather may specifically target phagocytic cells to promote survival of bacteria after the onset of innate immune defenses. This review will summarize key experiments performed over the past 10 years to reveal the function of these toxins.

## 1. Bacterial Protein Toxins with an Actin Crosslinking Domain

### 1.1. Overview of actin targeting toxins

Many toxins have been characterized that disrupt the eukaryotic actin cytoskeleton. These include fungal and marine small molecule toxins that bind to actin to prevent actin polymerization [1,2]. Other bacterial proteins toxins and effectors can alter the normal regulation of cytoskeletal assembly, by directly or indirectly modifying the activity of small Rho family GTPases [3]. Finally, toxins have been identified that covalently modify actin itself, for example by the addition of an ADP-ribosyl group that then prevents its incorporation into F-actin [4,5].

Studies over the past ten years have been directed toward characterizing a distinct mechanism to disrupt actin filament assembly by covalently crosslinking actin monomers. The crosslinks can be introduced repetitively to form long chains of dysfunctional actin that cannot be incorporated into F-actin filaments, resulting in irreversible destruction of the host cell cytoskeleton [6].

### 1.2. Discovery of the actin crosslinking domain of MARTX_Vc_

An actin crosslinking toxin was first discovered in current pandemic strains of the human diarrheal pathogen *Vibrio cholerae* [6]. When added to mammalian cells in culture, *V. cholerae* was shown to secrete a factor that induced rapid cell rounding, ultimately resulting in loss of all polymerized actin [6,7]. When actin in *V. cholerae*-treated cells was monitored by western blotting as a potential target for covalent modification, it was noted that all the monomeric actin in the cell disappeared within 180 min of addition of bacteria and actin was detected as higher weight molecular forms. The change in the electrophoretic mobility of actin corresponded to the formation of dimers, trimers, and higher order oligomers, revealing that actin in *V. cholerae*-treated cells had been crosslinked into chains [6].

This actin crosslinking activity depended upon the gene *rtxA*, the largest gene of the *V. cholerae* genome [6,7] and actin crosslinking activity was found to be widely present among both clinical and environmental isolates of *V. cholerae* [8,9,10,11]. The protein encoded by *rtxA*, now referred to as the Multifunctional, Autoprocessing RTX toxin of *V. cholerae* (MARTX_Vc_), is the founding member of a new family of toxins [12]. The MARTX toxins are a grouping of toxins that are all large in size (>350 kDa) and are secreted from the bacteria by dedicated Type I secretion systems. Conserved features of all MARTX toxins include N- and C-terminal glycine rich repeats and an autoprocessing cysteine protease. These conserved structural elements are proposed to function in toxin translocation to deliver one to five effector domains found in the central region of the toxins to the eukaryotic cell cytosol [12].

To identify the specific effector of MARTX_Vc_ responsible for actin crosslinking, subfragments of *V. cholerae* *rtxA* gene were ectopically expressed in epithelial cells as fusions to GFP and GFP-positive cells were visualized for rounding. Cells transfected with DNA corresponding to amino acids 1963-2375 of the *rtxA* gene (according to the original annotation by Lin *et al.* [7]) resulted in cell rounding and cells were confirmed to contain crosslinked actin by western blotting. This region was thus designated the actin crosslinking domain, or ACD [13], and it is now recognized as an effector delivered by autoprocessing of the large MARTX_Vc_ toxin [14].

### 1.3. Other proteins with actin crosslinking domains

**Figure 1:**
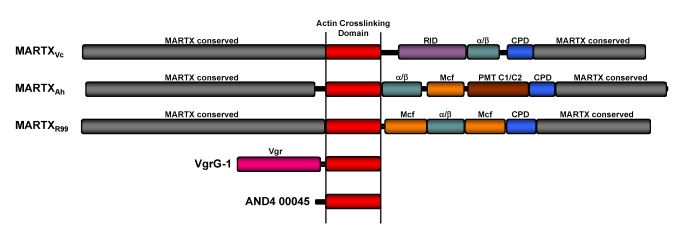
Actin crosslinking toxins. Scaled diagrams of the five known and putative actin crosslinking toxins. MARTX toxins of *V. cholerae* (MARTX_Vc_), *A. hydrophila* (MARTX_Ah_), and *V.vulnificus* Biotype 2 virluence plasmid pR99 (MARTX_R99_). Conserved MARTX repeat regions (grey) are predicted to converge at the eukaryotic plasma membrane to form a pore through which the centrally-located effectors are translocated. After translocation, the cysteine protease domain (CPD) initiates autoprocessing to release effectors, including the actin crosslinking domain (ACD-red), Rho-inactivation domain (RID-purple) [24], α/β hydrolase effector (α/β-green), Mcf conserved effector of unknown function (Mcf-orange), and *Pastuerella multocida* toxin conserved effector of unknown function (PMT C1/C2-brown)[12]. Note that MARTX_R99_ has a duplication of the Mcf effector. *V. cholerae* VgrG-1 is an effector of the *V. cholerae* VAS T6S system. The Vgr conserved domain (Vgr-pink) forms part of the translocation apparatus for transfer of the ACD across the phagosomal membrane after engulfment by phagocytes. *Vibrio* sp. AND4 hypothetical protein AND4_00045 is a putative stand-alone actin crosslinking toxin that is potentially an effector of an unknown transport apparatus.

Subsequent to the discovery of the ACD within MARTX_Vc_, two other MARTX toxins have been identified by protein sequence analyses that also carry ACD effectors. Genomic sequencing of *Aeromonas hydrophila* strain ATCC 7966T revealed this pathogen associated with disease in invertebrates and an emerging pathogen in humans has a gene for a MARTX toxin that includes an ACD effector [12,15]. By contrast, genomic sequencing of human pathogenic Biotype 1 *V. vulnificus* strains revealed they also encode a MARTX toxin [16,17], but this toxin was not associated with actin crosslinking and did not have an ACD [13,18]. However, an environmental *V. vulnificus* Biotype 2 strain isolated from a diseased eel was found to carry a gene for a second MARTX toxin on the pR99 virulence plasmid (MARTX_R99_) and this toxin included an ACD [19] (Figure 1). At this time, neither of these ACDs have been confirmed biochemically to be functional for actin crosslinking.

A second protein encoded by *V. cholerae* was also found to have an ACD [13]. This protein, VgrG-1, is a bacterial effector that is secreted from *V. cholerae* dependent upon a Type 6 secretion (T6S) system [20]. The first domain of the protein belongs to the VgrG family, a group of proteins required to form a structural element of the T6S apparatuses [21]. VgrG-1 is unusual among the Vgr proteins because it is an “evolved VgrG” with an attached second domain that is delivered to the cell cytosol and carries effector function, in this case, an ACD that has been demonstrated experimentally to have actin crosslinking activity [22] (Figure 1).

The final putative actin crosslinking toxin is hypothetical protein AND4_00045 identified in the draft genome of environmental *Vibrio* sp. AND4 isolated directly from the Andaman Sea in Thailand. Unlike the MARTX toxins or VgrG-1, this protein is a stand-alone actin crosslinking protein with no other associated domain for translocation [23]. Thus, if it is an effector used for pathogenesis of aquatic animals, it must be translocated directly to the eukaryotic cell cytosol, possibly by a Type III or T6S system also identified in the draft genome.

## 2. Characterization of the Actin Crosslinking Activity

### 2.1. ACD directly crosslinks actin

Early models of the mechanism of actin crosslinking recognized that crosslinking could occur either directly, in which the ACD is the catalytic enzyme, or indirectly, by activation of an unknown host cell protein with this activity [6]. To demonstrate direct action, the ACD portion of MARTX_Vc_ was purified as a fusion with the *N*-terminus of Anthrax Toxin Lethal Factor (LF_N_). LF_N_ has been shown previously to mediate cytosolic delivery of heterologous fusion proteins through pores formed by Anthrax Toxin Protective Antigen (PA) [25,26]. This approach facilitated pretesting the LF_N_ACD for biochemical activity in the cytosol of mammalian cells after delivery by PA, prior to initiating experiments to establish *in vitro* crosslinking [27].

Subsequent experiments showed that LF_N_ACD does crosslink actin both *in vivo* after delivery by PA, as well as *in vitro* when added to eukaryotic cell lysates. Further refinement of the crosslinking reaction revealed the *in vitro* reaction is catalytic and progresses to completion in less that 10 min. This crosslinking occurs under reaction conditions composed of only purified actin, ATP, and Mg^2+^, indicating that no other proteins or cellular co-factors are required for crosslinking of actin by the ACD; and thus, ACD itself is the catalytic enzyme [27].

### 2.2. Requirement of G-actin, ATP, and Mg^2+^ for actin crosslinking

Polymerization of monomeric G-actin into F-actin filaments is dependent upon hydrolysis of ATP [28] and occurs in the presence of 2 mM Mg^2+^. Hence, the requirement of Mg^2+^ and ATP during the *in vitro* crosslinking reaction was initially thought to indicate that actin must be polymerized to be crosslinked. However, it was shown that crosslinking *in vitro* progressed efficiently when actin was locked as monomeric G-actin by association with latrunculin or kabiramide C or by modification with tetramethyl red [29]. Similarly, actin crosslinking progressed *in vivo* after addition of MARTX_Vc_-producing *V. cholerae* to cells that were pretreated with cytochalasin or latrunculin to inhibit actin polymerization [27,30]. By contrast, stabilization of F-actin *in vitro* by the addition of phalloidin or *in vivo* using dolastatin 11 inhibited the efficiency of actin crosslinking [27,29]. These data indicated that G-actin, not F-actin, is the substrate for ACD in the crosslinking reaction. Furthermore, these data support that the requirement for ATP and Mg^2+^ during ACD-mediated actin crosslinking is not to drive actin polymerization.

**Figure 2:**
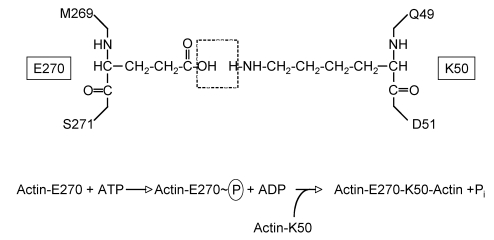
Crosslink introduced between actin side chains. Schematic shows E270 and K50 side chains originating from two different actin monomers with dashed box indicating H_2_O molecule lost during the ligation reaction. Lower equation depicts the predicted phosphotransfer based on the mechanism for formation of γ-glutamylcysteine from glutamic acid and cysteine.

Interestingly, by contrast to studies with MARTX_Vc_, crosslinking of actin in macrophages due to VgrG-1 was completely blocked by cytochalasin. However, this inhibition was not due to the ACD of VgrG-1 requiring polymerized actin instead of monomeric actin; rather it revealed a requirement for the bacteria to be phagocytosed before the VgrG-1 effector could be delivered to the cytosol by T6S [21]. Next, to account for the requirement of ATP and Mg^2+^ in the crosslinking reaction, it was considered if the ACD itself is an ATPase. During an *in vitro* crosslinking reaction, inorganic phosphate (P_i_) is released in a dose dependent manner such that one molecule of ATP is used for each crosslink introduced. This ATP hydrolysis is not due to polymerization of actin, but is an essential aspect of the crosslinking reaction itself [29] (see Figure 2).

### 2.3. Most G-actin binding proteins do not inhibit the crosslinking reaction

It is well-recognized that in the living cell, G-actin does not exist in a free form and is typically bound to one of the several actin binding proteins (ABPs) [31]. Remarkably, prior association of actin with the common ABPs profilin, thymosin-β4, and gelsolin did not have any noticeable effect on actin crosslinking by ACD. Among the ABPs tested, only DNaseI completely blocked the formation of actin oligomers, and cofilin partially inhibited. These data show that *in vivo*, crosslinking of actin by MARTX_Vc_ likely progresses without interference of most ABPs [29].

Furthermore, actin crosslinking can occur when actin is previously crosslinked to itself. As G-actin is the substrate for crosslinking, higher order oligomers could arise by continued addition of G-actin monomers to longer chains or by the joining of oligomers. It was found that actin dimers disappear during crosslinking at ~4 fold slower rate than the monomers. This result indicated that although ACD-crosslinked oligomers can be further joined to form higher order species, the efficiency of the reaction diminishes with increased size of oligomers [29]. Thus, the formation of long crosslinked actin chains may occur predominantly by addition of monomers to linked oligomers.

## 3. The Chemical Nature of the Crosslink

The chemical crosslink introduced into actin has been solved and shown to be an isopeptide linkage between glutamate 270 on one actin monomer with lysine 50 on a second actin monomer (Figure 2). 

No other crosslinks are introduced and mutation of these residues within actin prevented crosslinking entirely, both *in vitro* and *in vivo* [32]. The crosslinked residues occur in flexible loops of actin in regions isolated away from binding sites for latrunculin, kabiramide C, and ABP binding sites. Lysine 50 is a residue on the DNaseI binding loop accounting for the lack of crosslinking when actin was pre-associated with DNaseI [29,32].

As seen in the crystal structure of the dimerzied form of crosslinked actin (PDBID: 3CJC), the two actin molecules are tilted with respect to the normal alignment of polymerized actin. When added to a crosslinking reaction, the altered structure cannot facilitate addition of new monomers such that dimerized actin competes with monomeric actin in F-actin growth and halts further polymerization [32]. Thus, actin crosslinking ultimately rounds cells both by actively blocking growth of actin filaments and by depleting the G-actin pool.

## 4. The Catalytic Mechanism Is Similar to Glutamate Synthetases

Given the novel nature of the crosslink in actin, it is not surprising that little information regarding the mechanism of catalysis could be derived using bioinformatic analyses. 

**Figure 3:**
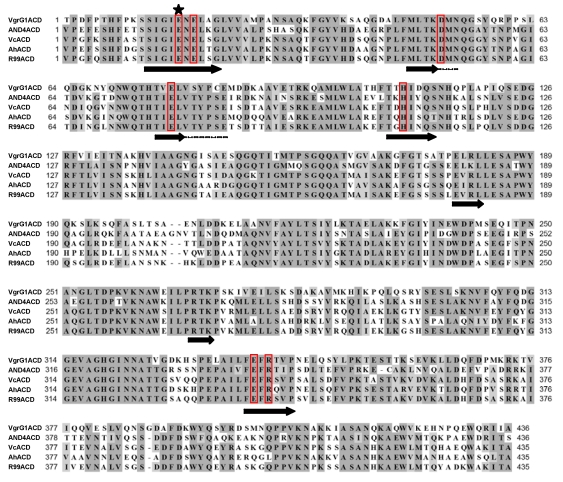
Alignment of ACD amino acid sequences from actin crosslinking toxins. Alignment was generated with CLUSTALW using MacVector 9.5.2 software based on sequences available at NCBI PubMed with accession numbers: *V. cholerae* VgrG1 ACD (NP_231059.1); *Vibrio* sp. AND4_00045 ACD (ZP_02197558.1); *V. cholerae* (Vc) MARTX ACD (AAD21057.1); *A. hydrophila* (Ah) MARTX ACD (YP_855898.1); and *V. vulnificus* Biotype 2 virulence plasmid pR99 MARTX ACD (YP_001393065.1). Red boxes indicate residues identified by genetic analysis as important for crosslinking with the star indicating the glutamic acid required for phosphotransfer. Black arrows represent predicted beta-strands that might form a glutamine synthetase ligase-like active site (see Figure 4).

**Figure 4:**
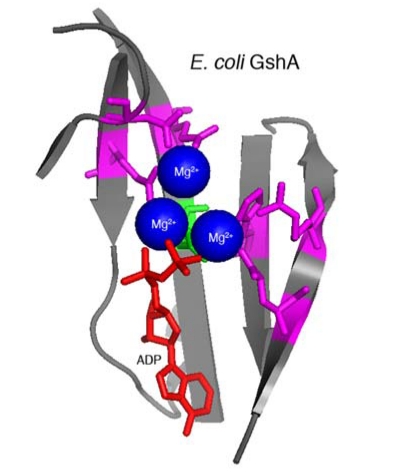
Structural model of E. coli GshA. E. coli GshA (PDB 1VA6; [34]) active site is composed of a beta-sheet (grey) that contributes amino acid side chains that bind three Mg^2+^ ions (blue) and ATP (ADP in crystal structure, red). The active site shares conserved residues with ACD amino acids demonstrated to be important for crosslinking activity (magenta). The essential ACD glutamate residue (green) is likely required for phosphotransfer during the ligation reaction as shown in Figure 2. Model prepared using MacPyMol (Delano Scientific) based on information from Geissler et al. [33].

Protein alignments using the primary peptide sequence of the ACDs identified only that ACD proteins are highly conserved, but showed no homology to other known or hypothetical proteins, suggesting actin crosslinking by an ACD may be restricted to the *Vibrionaceae* bacteria (Figure 3). 

Without sequence-based information to inform research strategies, the catalytic mechanism was probed by a random genetic approach to identify essential amino acids within the ACD [33]. This analysis revealed that glutamic acid 1990 of MARTX_Vc_ is absolutely essential for actin crosslinking while mutation of six other residues resulted in decreased actin crosslinking efficiency. No other important residues were identified. A structural alignment algorithm revealed these residues could be mapped against the conserved catalytic site of proteins in the glutamine synthetase family of ATP-dependent protein ligases. Modeling ACD using the crystal structure of γ-glutamylcysteine synthetase (GshA) from *Escherichia coli* [34] revealed that E1990 likely participates in the transfer of the γ-phosphate from ATP to actin glutamate 270 side chain to activate the ligation reaction with lysine 50, resulting in release of P_i_ (Figure 2). Thus, actin crosslinking to link glutamate to lysine occurs by a ligation reaction similar to that required to attach ammonia to glutamate to form glutamine or cysteine to glutamate to form γ-glutamylcysteine [33]. Given the strong conservation of important residues among the five identified ACDs (Figure 3), it is reasonable to predict that all ACDs will crosslink actin by an identical mechanism.

## 5. Phagocytic Cells as the Primary Target of ACD

The predominance of work studying the action of actin crosslinking has been conducted using epithelial cells in culture. However, the role of these toxins in disease progression is unclear because, particularly for cholera disease, there is limited epithelial damage. Recent studies have been directed toward understanding whether actin crosslinking toxins more specifically target innate immune cells. MARTX_Vc, _in conjunction with other secreted factors, has been demonstrated to be important to prevent clearance of the bacteria from the small intestine [35,36]. This effect of toxin expression on the ability of the bacteria to persist suggests MARTX_Vc_ effectively blocks clearance by slowing or completely deactivating phagocytosis of the bacteria. Consistent with this model, the virulence plasmid that encodes MARTX_R99_ promotes survival of *V. vulnificus* Biotype 2 strains in eel whole blood suggesting it may inactivate phagocytes [19]. Furthermore, the actin crosslinking Type VI secretion effector VgrG-1 from *V. cholerae* crosslinks actin within phagocytic macrophages and amoebae, but not epithelial cells [20,21]. Finally, *A. hydrophilia* has been demonstrated to evade phagocytosis, although this may be as much or more related to its T6S system and associated actin ADP-ribosylating effector [5,37]. Thus, it seems possible that the function of the actin crosslinking toxins could be to directly block bacterial engulfment by phagocytic cells to evade clearance from the site of infection. This has been experimentally demonstrated for the function of VgrG-1 proteins and infection data suggest other actin crosslinking toxins may function in similar capacity to facilitate colonization and disease [38].
